# The emerging role of machine learning-based methods in cancer classification using microRNA

**DOI:** 10.1016/j.bbrep.2026.102506

**Published:** 2026-02-14

**Authors:** Zeinab Tariri, Mehdi Goodarzi, Atieh Nouralishahi, Malihe Sagheb Ray Shirazi, Meysam Mohammadikhah, Azita Sadeghzade, Hossein Gandomkar, Ehsan Maghrebi-Ghojogh

**Affiliations:** a Department of Microbiology, School of Biological Sciences, Islamic Azad University Tehran North Branch, Tehran, Iran; b Islamic Azad University, Sepidan Branch, Shiraz, Fars, Iran; c Faculty of Electrical, Computer, IT and Biomedical Engineering, Islamic Azad University, Qazvin campus, Qazvin, Iran; d Student Research Committee, West Hormozgan School of Medical Sciences, Hormozgan University of Medical Sciences, Bandar Abbas, Hormozgan, Iran; e Department of Oral and Maxillofacial Surgery, School of Dentistry, Alborz University of Medical Sciences, Karaj, Alborz, Iran; f Oral and Dental Disease Research Center, Department of Oral and Maxillofacial Medicine, School of Dentistry, Shiraz University of Medical Sciences, Shiraz, Iran; g Department of Surgical Oncology, Tehran University of Medical Medicine, Tehran, Iran; h Pharmaceutical Sciences Research Center, Faculty of Pharmacy, Mazandaran University of Medical Sciences, Sari, Iran

**Keywords:** microRNA, Machine learning, Cancer, Cancer classification

## Abstract

Early detection and accurate classification of cancer are crucial to improving patient outcomes. Diagnosis and classification of tumors using conventional methods remains challenging. MicroRNAs (miRNAs) are potential biomarkers for accurate tumor classification and differentiation of tumor subtypes. In cancer progression, miRNAs act as oncogenes or tumor suppressors to regulate gene expression. As a result of their stability in bodily fluids such as blood, urine, and saliva, they are ideal for non-invasive diagnostic procedures. Machine learning (ML) models can identify discriminative miRNAs for various cancers, such as breast, lung, colorectal, and kidney cancers. The integration of ML with miRNA data has demonstrated significant potential for differentiating cancerous tissues from normal tissues and identifying clinically relevant biomarkers. For instance, techniques such as feature engineering and selection, including recursive ensemble selection and miRNA-mRNA network analysis, have been shown to enhance both model accuracy and interpretability. Methods based on Random Forest (RF) and Support Vector Machines (SVM) have successfully classified breast cancer subtypes, and miRNA signatures from fecal samples have been highly effective in diagnosing colorectal cancer. Furthermore, deep learning and neuro-fuzzy systems support kidney cancer analysis, highlighting miRNA-driven ML's role in cancer diagnostics and personalized treatment. This review illustrates the transformative potential of miRNA-driven ML models for advancing cancer diagnostics and enabling personalized treatment strategies.

## Highlights

• Early cancer detection and precise classification are key to better outcomes.
• Standard diagnostics still struggle to distinguish cancer types and subtypes.
• MicroRNAs influence cancer progression and are detectable in body fluids.
• MicroRNAs show strong potential as biomarkers for tumor classification.
• Machine learning helps identify microRNAs that differentiate cancer types.

## 1 Introduction

Cancer is among the main causes of mortality worldwide. Recently, there have been notable advancements in the detection and treatment of tumors [1]. However, early detection and proper cancer classification remain crucial for enhancing outcomes and decreasing recurrence and death rates in cancer patients. The International Union for Cancer Control (UICC) and the World Health Organization (WHO) define characteristics such as origin, type, grade, histotype, and spread throughout the body to diagnose and classify tumors [2]. According to morphological, epidemiological, and biological data, these classifications have been established to facilitate management decisions of cancer patients [3]. To apply these cancer classification guidelines, the appropriate skills and instrumentation for clinical pathology, diagnostic imaging, and molecular testing are required. As a result, there are challenges and variations in the implementation of cancer classification guidelines worldwide today. This highlights a primary need to enhance and expand diagnostic techniques, particularly within the realm of molecular testing, to facilitate more accurate cancer detection and classification. Biological markers such as microRNAs (miRNAs) are used in many malignancies to classify certain tumor types based on the absence and presence of specific genetic abnormalities [4,5].

As demonstrated by microarray analysis and sequencing data of various cancer types, abnormal miRNA expression is one of the primary causes of cancer [6]. Besides, miRNAs have demonstrated significant potential as biomarkers in elucidating cancer subtype differentiation and survival. The identification of key miRNAs and the selection of the most discriminative miRNAs as biomarkers are the primary objectives of scientific research aimed at differentiating cancer subtypes and stages. On the other hand. The limited access to high-throughput miRNA data and the small number of known miRNAs were the biggest challenges faced by researchers. High-throughput methods, such as microarrays [7] and RNA-seq [8], have generated large amounts of miRNA data in a short period of time and at minimal cost for various cancer samples. The Genomic Data Commons (GDC) and The Cancer Genome Atlas (TCGA) are among the databases that provide significant miRNA-seq datasets for at least 33 cancer types [7].

Machine learning (ML)-based algorithms have been developed to classify various cancers by using miRNA-seq datasets [8]. These mathematical approaches can learn from data and make predictions or decisions. Therefore, a small set of miRNAs with high potential in distinguishing cancer types and subtypes can be identified. The importance of miRNAs in cancer typing, the ML algorithms used to classify cancer, and the discovery of discriminative miRNAs in cancer-related miRNA-seq datasets are all addressed in this comprehensive review.

## 2 Role of miRNAs in cancer diagnosis and classification

### 2.1 Overview of miRNA biogenesis

**Fig. 1:**
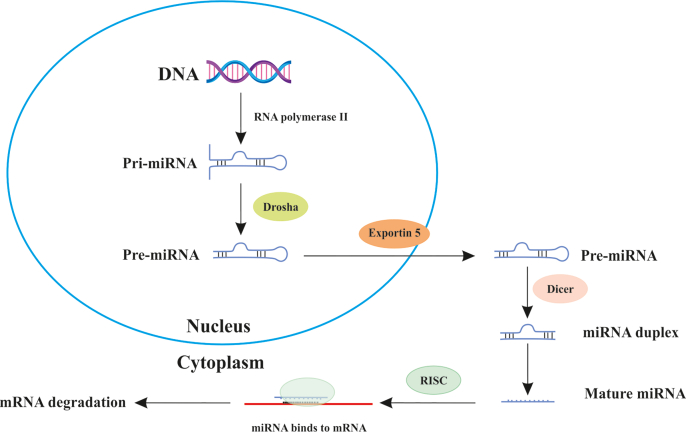
The miRNA biogenesis process.

MiRNAs are promising candidates for cancer biomarkers, as they can provide valuable information about the molecular characteristics, biological behavior, and clinical outcome of tumors [9]. This short non-coding RNA (18-22 nt) is usually transcribed from sets of related genes, where each gene either already has a promoter or may be intragenically generated from spliced parts of genes that code for proteins [10]. RNA polymerase II initiates the transcription of miRNAs as a primary miRNA (pri-miRNA) [11] (Fig. 1). DGCR8 (DiGeorge Syndrome Critical Region 8) and Drosha, a RNase III enzyme, are used to cleave pri-miRNA into 70-80 nt hairpin precursors (pre-miRNAs) [12]. DGCR8, a protein with 773 amino acids, interacts with double-stranded RNA and creates a micro complex with the Drosha enzyme. The pre-miRNAs are transported to the cytoplasm via Exportin-5 (XPO-5), where they undergo cleavage by Dicer into mature miRNAs. The miRNAs control their target genes by binding to complementary sequences, frequently discovered in the 3′ untranslated region (UTR) of the target mRNA, leading to mRNA degradation or inhibition of translation [13]. Their significant role in regulating biological processes in cancer makes miRNAs valuable for diagnosis, treatment, and cancer typing.

### 2.2 MiRNA-based cancer typing

Cancer classification involves categorizing various forms of tumors based on their histological features, site of origin, clinical behavior, and molecular biomarkers.

In tumors, miRNAs may have significant impacts on cancer formation and progression by functioning as either tumor suppressors or oncogenes [14]. Besides, they are detectable in several bodily fluids, including blood, saliva, urine, and cerebrospinal fluid [15]. These circulating miRNAs are stable, specific, and easily measurable, making them promising biomarkers for cancer diagnosis and categorization. The ability to detect miRNAs from biological fluids, coupled with the development of high-quality measurement methods, makes them highly promising for future diagnostic applications [16].

The classification of certain malignancies is important in elucidating tumor processes and establishing treatment strategies. Specific miRNAs that are expressed differently can be used in cancer typing. For instance, the precise distinction between squamous cell carcinoma (SCC) and adenocarcinoma (ADC) subtypes in non-small lung cancer (NSCLC) has implications for the choice of therapeutic strategies [17]. In contrast to healthy individuals, those with SCC and ADC have higher levels of miR-486-5p and miR-16-5p [18]. While the miR-9-5p expression is unchanged in healthy controls and overall NSCLC patients, it shows a substantial decrease in ADC patients rather than SCC patients [18]. Furthermore, using RNA sequencing, Jin et al. identified several miRNAs that were differently expressed between ADCs and SCCs [19]. In NSCLC plasma exosomes, miR-320b, miR-181-5p, and miR-361-5p were upregulated. ADC patients had 10-fold higher levels of miR-361-5p and miR-181-5p than SCC patients, while SCC patients had 10-fold higher levels of miR-320b than ADC patients. In breast cancer, it is important to subtype heterogeneous types for therapeutic treatment. Indeed, tumors that express Estrogen receptor (ER), Progesterone receptor (PR), or Human epidermal growth factor receptor 2 (HER2) can be positively targeted by specific therapies. Traditional chemotherapy or radiation treatment is often used to treat individuals with triple-negative breast cancer (TNBC), which does not express any of these receptors [20]. TNBC is associated with advanced stages and a poorer prognosis. In a study by Shin et al., it was noted that the levels of miR-21-5p, miR-16-5p, and miR-199a-5p were lower in the tumor tissues and plasma of TNBC patients than those of non-TNBC and healthy individuals. In addition, elevated levels of miR-373-3p can be related to TNBC and ER/PR-negative tumors [21].

One of the main challenges in using miRNAs as cancer biomarkers is to determine the smallest subset of miRNAs that has great potential in discriminating between cancer type and subtype. The reason for this is the large number of miRNAs in the human genome, and the fact that many of them have abnormal expression in various cancers [22]. Moreover, the expression of miRNAs can be affected by various factors, such as tissue origin, tumor stage, genetic mutations, environmental exposures, and treatment effects [23]. Identifying a universal and specific set of miRNAs for accurately diagnosing and classifying various cancer types is challenging. Researchers have used approaches including bioinformatics analysis, ML algorithms, and experimental validation to identify the most significant miRNAs for cancer typing and address these challenges [24,25].

## 3 ML approaches for cancer classification

Several large-scale cancer genomics programs, including The Cancer Genome Atlas Program (TCGA), have been inspired by next-generation sequencing (NGS) and array-based whole-genome profiling methods, which allow rapid molecular analysis of cells or tissues [7,26]. These programs aim to characterize the genetic, epigenomic, and transcriptional characteristics of patients with various types of cancer. The large amount of data generated by these approaches has resulted in the development of several analytical methods, including ML algorithms (Table 1).

**Table 1:** Different supervised ML-based algorithms for cancer classification and their main advantages and disadvantages.

Algorithm	Pros	Cons	Ref
KNN	• Simplicity and ease of implementation • Versatility • No training periods • Non-parametric nature	• Computationally intensive • Sensitivity to irrelevant features • Need for optimal K-value	[27]
Logistic Regression	• Efficiency with small datasets • Feature selection • Prediction of probabilities	• Finding liner relationships • Prone to overfitting when using large-scale datasets • Difficult interpretation when using high-dimensional data sets • Low flexibility and scalability	[28]
Linear regression	• Robustness and flexibility • Simple data preparation • Not time consuming • Ease of interpretability • Sensitive to outliers	• Not ideal for classification • Finding liner relationships	[29]
SVM	• Has the best performance when the clear separation between classes is available • High efficacy with high-dimensional data • Modeling non-linear decision boundaries by using different kernel functions	• Prone to overfitting or underfitting when using inadequate parameters • Time- consuming process • Low transparency and interpretability	[30,31]
DT	• Flexible • Non-linear • Useful for data exploration • Interpretable • Non-parametric	• Computationally expensive • Overfitting • Unstable to changes in the data	[32,33]
RF	• Increase model accuracy • Elevated model stability • Evaluate non-linear connections between features • Handling a combination of numerical and category features • Simple to understand	• High cost of computation • It may be slow and ineffective for real-time predictions	[34,35]

**Fig. 2:**
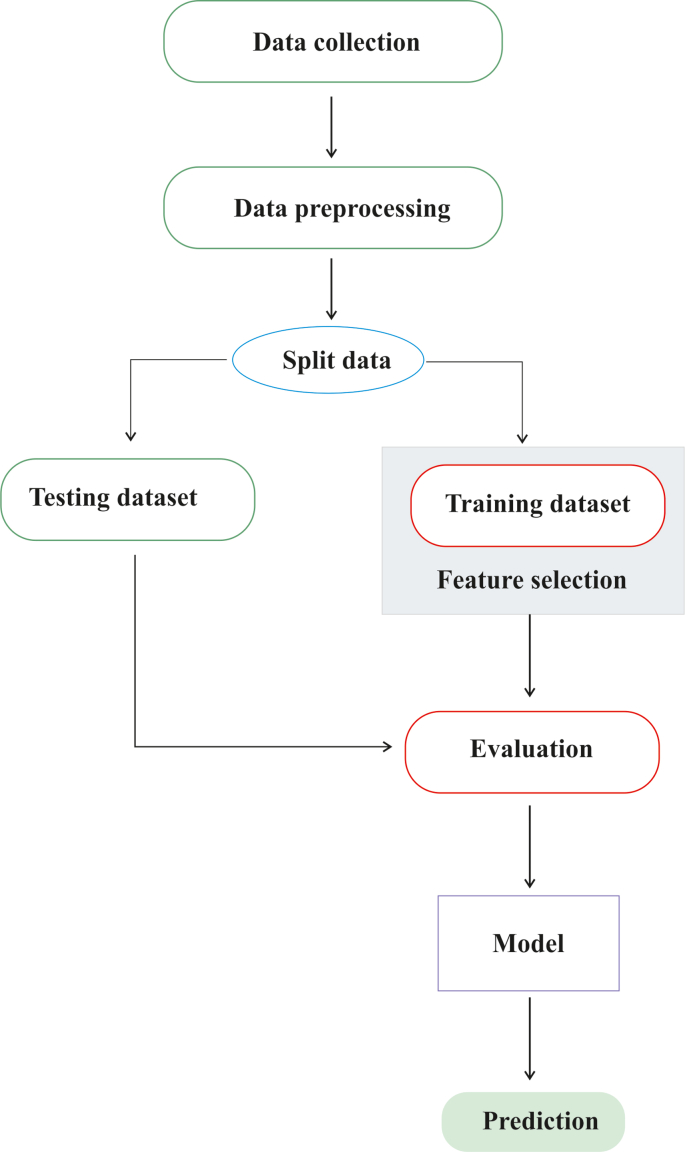
The workflow of ML-based approach for predication from raw data.

The ML process involves data collection and preprocessing, followed by data splitting, feature selection, evaluation, and modeling (Fig. 2). ML has become essential for analyzing miRNA expression data in cancer. Each dataset describes a sample by recording the expression level of each miRNA. A single sample is represented as a long vector, with each variable corresponding to the expression level of a specific miRNA, often resulting in hundreds or even thousands of variables per sample [36]. This high-dimensional scenario can overwhelm traditional statistical methods, leading to overfitting. In contrast, ML methods are specifically designed to handle such challenges, stabilizing the learning process and identifying the most informative miRNAs. ML algorithms can learn from intricate patterns in the data, enabling them to differentiate between various sample classes, such as cancer versus normal or different tumor subtypes [37]. These algorithms can be broadly categorized into two main types: supervised learning, where models are trained on labeled miRNA profiles to identify patterns that differentiate predefined tumor classes, and unsupervised learning, where models analyze unlabeled data to reveal intrinsic groupings or substructures, potentially representing novel cancer subtypes or biological states. Over the past decade, a range of ML techniques have been employed for miRNA-based cancer classification. Supervised methods, including SVM, RF, and deep neural networks, alongside unsupervised approaches such as clustering and principal component analysis (PCA), have played significant roles in advancing biomarker discovery and tumor stratification [8,38,39].

In the following sections, we provide an overview of the primary classes of ML algorithms utilized in miRNA-driven cancer classification, their operational mechanisms, and their applications across various cancer types. We examine both supervised and unsupervised learning approaches in miRNA-based cancer classification, exploring their applications to specific cancers, including breast, lung, colorectal, liver, and prostate cancers. We also analyze their roles in tumor subtyping and compare their performance, strengths, and limitations based on findings from recent studies.

### 3.1 Preprocessing

Data preprocessing is a crucial step in developing ML models, particularly when working with high-throughput genomic data, such as miRNA expression profiles [40]. Raw miRNA datasets often have high dimensionality, sparsity, and noise, typically containing thousands of features but a relatively small number of patient samples [41]. Without proper preprocessing, these issues can lead to overfitting, reduced generalizability, and biased classification outcomes. Therefore, preprocessing not only ensures data quality and consistency but also enhances the ability of ML models to identify biologically meaningful patterns, which is essential in cancer classification tasks where subtle molecular signatures define disease subtypes.

One of the most widely used sources for miRNA data in cancer research is The Cancer Genome Atlas (TCGA), which provides large-scale, publicly accessible RNA sequencing data across multiple cancer types, including kidney cancer subtypes [42]. In addition to TCGA, other consortia, such as TARGET (Therapeutically Applicable Research to Generate Effective Treatments), also provide curated miRNA datasets for specific malignancies, including Wilms tumor and rhabdoid tumor. Beyond these repositories, miRNA expression profiles can also be generated through experimental methods such as microarray hybridization, quantitative RT-PCR, or next-generation sequencing (NGS) in laboratory studies [43,44]. While experimental approaches allow researchers to design disease-specific cohorts, public databases such as TCGA and TARGET remain invaluable for developing and benchmarking ML models due to their standardized protocols and large sample sizes.

The preprocessing of miRNA data typically involves several steps to prepare raw sequencing data for ML analysis [45]. These steps include downloading expression matrices, converting file formats for computational use, aligning miRNA data with clinical labels, and splitting datasets into training, validation, and testing cohorts to ensure unbiased model evaluation. A crucial step is data normalization, which reduces technical variability and ensures that features are comparable across samples. Z-score normalization is commonly employed for this purpose. In the study by Pirmoradi et al. [46], preprocessing followed this standard workflow: raw miRNA data from TCGA/TARGET were converted into MATLAB-readable files, matched with subtype labels, partitioned into training (70%), validation (10%), and testing (20%) sets, and finally normalized using Z-score scaling. These steps ensured that the subsequent feature selection and classification processes were applied to high-quality, standardized data, thereby improving both model robustness and clinical relevance.

### 3.2 Feature engineering

Feature engineering is a pivotal step in the ML pipeline, particularly when working with high-dimensional biological data, such as miRNA expression profiles [47]. In contrast to preprocessing, which focuses on cleaning and standardizing raw data, feature engineering creates novel, biologically relevant variables that better capture the underlying mechanisms. These variables enhance predictive performance. In cancer classification, raw miRNA expression values often fail to reflect the complexity of regulatory interactions and disease heterogeneity [48]. Transforming raw data into meaningful features, such as network connectivity measures, improves model interpretability, reduces noise, and uncovers latent structures. This step is essential for building robust and generalizable models. It not only improves classification accuracy but also sheds light on the mechanistic roles of miRNA in tumor progression and clinical outcomes.

Constructing a dysregulated miRNA–mRNA network (DMTN) is an advanced feature engineering approach that surpasses the limitations of raw expression data [49]. Rather than isolating each miRNA as an individual feature, this approach models cancer as a network of disrupted regulatory interactions. High-confidence miRNA–mRNA pairs are identified from experimentally validated databases, such as ENCORI/starBase, which incorporate CLIP-seq data [50]. For each miRNA–mRNA pair, expression correlations are calculated separately for tumor and normal samples, and a dysregulation score is derived from the difference between them. Pairs with significantly altered correlations are retained to construct the network. The resulting DMTN not only identifies differentially expressed miRNAs and mRNAs but also reveals how their regulatory relationships are rewired in cancer. From this network, quantitative features such as out-degree, co-regulation patterns, and neighbor overlaps are derived. These graph-based attributes enrich the feature space for ML models, offering a more biologically informed representation. This approach effectively transforms complex interaction patterns into measurable variables, enhancing both predictive performance and mechanistic understanding of miRNA roles in tumor biology. However, while the network-based preprocessing clearly enhanced performance and interpretability, it also introduced certain challenges. The reliability of such networks depends heavily on the accuracy of target prediction databases, which are often incomplete or biased toward well-characterized miRNAs [51]. Moreover, network construction can be computationally demanding and may reduce reproducibility across datasets from different platforms or cancer types [52]. These limitations suggest that, although effective, network-based preprocessing should be complemented with independent validation and cross-cohort testing before clinical translation.

### 3.3 Feature selection in miRNA-based cancer classification

Following feature engineering, the next critical step in the ML pipeline is feature selection [53]. In miRNA-based cancer classification, accurately distinguishing between cancer types or predicting their subtypes requires selecting the most informative and relevant features, which is essential for achieving optimal model performance. Without proper selection, irrelevant, redundant, or noisy features can increase computational costs, lower accuracy, and heighten the risk of overfitting. Feature selection addresses these issues by focusing on attributes that most impact cancer classification or subtyping, improving model efficiency, generalization, and robustness [54]. In miRNA-based cancer classification, selecting the most relevant miRNAs not only boosts predictive accuracy but also enhances biological interpretability, as retained features are often directly associated with mechanisms of tumor progression and clinical outcomes.

**Fig. 3:**
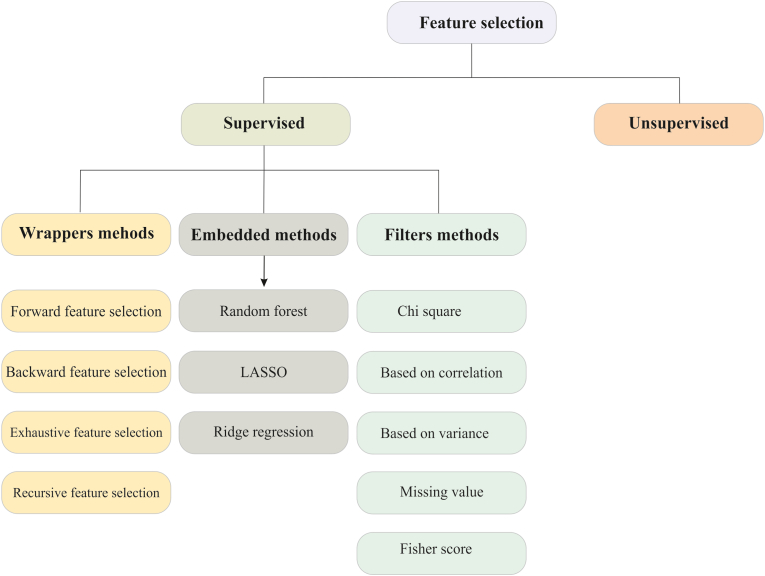
**Different feature selection approaches.** Filter method that evaluates each feature separately and assigns an appropriate score based on the relevance of features; Wrapper method that accounts for the interplay among features and uses a classifier to measure the performance of each feature subset; Embedded method that performs feature selection during the training process and examine the interplay among features.

Feature selection methods are generally categorized into three main types: wrapper methods, which search for the optimal subset of features by repeatedly training a classifier, but are computationally expensive and prone to overfitting [55]; embedded methods, which perform feature selection during the training phase of a specific model, offering tighter integration but with model dependency [56]; and filter methods, which rank features individually based on statistical criteria, making them classifier-independent and computationally efficient [57] (Fig. 3). In miRNA data, which is high-dimensional and sparse, filter methods are particularly advantageous as they avoid the heavy computational cost of wrapper approaches and the algorithmic constraints of embedded methods. Among various filter-based approaches, dispersion measures are commonly used to quantify how well a feature differentiates between classes. While measures such as variance and Mean Absolute Difference (MAD) can be effective, they often become less reliable in sparse, high-dimensional data. Similarly, information-theoretic measures (e.g., entropy or mutual information) rely on probability density estimation, which can be biased when the dataset is small relative to its dimensionality [58]. To address these limitations, a recent study introduced the Arithmetic Mean–Geometric Mean (AMGM) ratio as a dispersion-based criterion for assessing feature relevance [46]. The AMGM measure calculates the ratio between the arithmetic and geometric means of a feature's distribution, with higher ratios indicating greater discriminative power. Importantly, AMGM is computationally simple, less biased in sparse data, and particularly well-suited to miRNA datasets with thousands of features but relatively few samples. By applying AMGM, the authors identified a compact set of 60 miRNAs with the highest discriminant ability across kidney cancer subtypes.

To address the challenge of high-dimensional miRNA datasets, recursive ensemble feature selection has been applied to enhance the accuracy of cancer classification [59]. This approach aimed to reduce the data dimension by systematically selecting the most informative miRNAs while eliminating irrelevant or redundant features. A heterogeneous ensemble of classifiers, including SVM, RF, and Gradient Boosting, was used to aggregate feature rankings from multiple algorithms, thereby minimizing biases introduced by any single classifier. The recursive process systematically eliminates features with the lowest scores, ending when the model's classification accuracy falls below a predefined threshold. This robust and reliable ensemble strategy yielded a compact set of five miRNAs (hsa-let-7a, hsa-miR-23b, hsa-miR-122, hsa-miR-708, hsa-miR-200c) that significantly enhanced classification performance, achieving an accuracy of over 90%. In another investigation, recursive ensemble feature selection was applied using multi-method feature selection techniques, such as MIM (Mutual Information Maximization), mRMR (Minimum Redundancy Maximum Relevance), and CMIM (Conditional Mutual Information Maximization), to assess feature relevance [60]. The scores from these methods were aggregated to identify the most relevant miRNAs for the target classes. This approach, similar to the recursive ensemble method, focused on maximizing feature relevance to the target class while minimizing redundancy; however, it relied on feature selection techniques rather than classifiers to determine feature importance.

Employing several independent feature selection strategies and prioritizing their overlapping results is a robust method for identifying reliable features. The study by Kim et al. demonstrates the performance of two feature selection strategies through a comparative analysis of miRNA-seq datasets [61]. Two feature selection strategies were employed to identify the most informative miRNA genes for cancer classification: the Fisher score and Infinite Feature Selection (IFS). The Fisher score, a supervised method, prioritizes features with the largest differences between classes relative to their within-class variance, making it straightforward and computationally efficient for distinguishing tumors from normal samples [62]. In contrast, IFS, an unsupervised, graph-based approach, evaluates feature relevance by considering its relationships across all possible feature subsets, capturing more global and potentially hidden dependencies among genes [63]. The comparative analysis showed that while both methods effectively highlighted cancer-related markers, IFS tended to select features that were more stable across repeated experiments, whereas the Fisher score often identified well-known discriminative genes with strong class separation. As a result, the Fisher score is more transparent and interpretable, while IFS offers a broader and potentially more robust selection, particularly in high-dimensional omics datasets. Three miRNAs, miR-145, miR-200c, and let-7c, were consistently identified by both the Fisher score and IFS. MiR-145 and let-7c were downregulated in tumor tissues compared to normal tissues, while miR-200c was upregulated in tumors [64,65].

### 3.4 ML-based algorithms in cancer classification via determining discriminative miRNAs

The use of ML techniques in cancer classification, particularly with miRNA data, marks a significant advancement in biomedical research. MiRNAs, as key regulators of gene expression, provide valuable insights for differentiating between cancerous and normal tissues and identifying molecular cancer subtypes. However, the complexity and high dimensionality of miRNA datasets, along with the variability of cancer types, present challenges for traditional diagnostic approaches. ML algorithms are effective at analyzing large datasets and uncovering hidden patterns, making them essential for overcoming these obstacles. By integrating diverse ML models, researchers can improve cancer classification accuracy and reliability while enhancing results interpretability, enabling more informed clinical decisions (Table 2, Table 3). By advancing these technologies, we will be able to develop precise and personalized treatment strategies that ultimately lead to improved patient outcomes. MiRNA-based cancer classification based on ML holds great potential for transforming cancer diagnosis and treatment.

**Table 2:** The ML-based algorithms using for classifying cancer different type and subtypes.

Cancer	Data	Feature tool	Models/Algorithm	Partitioning data	Accuracy	Ref
Breast	TCGA	PCA	Rpart, RF and Treebag	Training: 70%	-	[66]
				Testing: 30%		
	TCGA	Infinite feature selection (IFS) and Fisher score	SVM, KNN, and RF	Training: 60%Testing: 40%	SVM and KNN = 96%,RF = 90%	[61]
	miRbase	Chi-Squared (CHI2), Information Gain (IG) and Least Absolute Shrinkage and Selection Operation (LASSO)	SVM and RF	Training: 90%	99%	[67]
				Testing: 10%		
	Serum miRNA expression (qRT-PCR, 84 miRNAs)	miRNA PCR Array + preprocessing (scale, center)	Linear SVM	10-fold cross-validation (70% training, 30% testing in each fold)	Luminal A: 100%, Luminal B: 100%, TNBC: 100%, HER2+: 95%	[68]
Kidney	TCGA and TARGET projects	NCA	LSTM	Training: 80%	96%	[69]
		AMGM	SOFNN	Testing: 20%	93.2%	[46]
Colorectal	TCGA	-	SVM and RF	Training: 70%	76-79%	[70]
				Testing: 30%		
	Fecal miRNA (small RNA-seq, multicenter, >1270 samples)	Filter + Embedded feature selection (k-best strategy)	Gradient Boosting, Logistic Regression, RF	Training: 70%	AUC: up to 0.96 (validation cohort)	[71]
				Testing: 30%		
	Fecal miRNA	Feature engineering (exponential, logarithmic, idempotent functions)	LDA	Training: 70%	Training: Accuracy 96.5%, Sensitivity 100%, Specificity 93.5%, AUC = 0.986.	[72]
				Testing: 30%		
Hepatocellular carcinoma	qPCR and TCGA	-	RF, LR, GBC and ANN	Training: 80%	RF = 91%	[73]
				Testing: 20%	GBC = 89%	
					ANN = 86%	
					LR = 84%	
	TCGA	Forward wrapper approach	RF, DT and SVM	-	RF = 98.2%	[74]
					SVM = 97%	
					DT = 83.1%

**Table 3:** Distinguishing miRNAs determined via various ML-based approaches.

Cancer	Models	Distinguishing miRNAs			Ref
		miRNA	Level	Tumor/Different subtypes	
Breast	Rpart, RF and Treebag	miR-139	Down	Tumor	[66]
		miR-183	Up		
		miR-4728	Up	HER2	
		miR-190b	Up	Luminal	
		miR-190b	Down	Basal	
	SVM, KNN, and RF	miR-145	Down	Tumor	[61]
		miR-200c	Up		
		let-7c	Down		
	RF and SVM	miR-10b	-	Tumor	[67]
		let-7c			
		miR-145			
	Liner SVM	miR-23b, miR-29a, miR-181c/d, miR-155		Luminal A	[68]
		miR-148a, let-7d, miR-92a, miR-34c		Luminal B	
		miR-125b, miR-134, miR-98, miR-143, miR-138		HER2+	
		miR-17, miR-150, miR-210, miR-372		TNBC	
Kidney	LSTM	35 miRNAs (let-7a-1, let-7a-2, let-7a-3, let-7b, let-7c, ….)		Renal Clear Cell	[46]
				Renal Papillary Cell	
				High-Risk Wilms Tumor	
	SOFNN	60 miRNAs (miR-7111, miR-584, miR-20b, …)		Kidney Chromophobe	[69]
				Rhabdoid Tumor	
Colorectal	RF and SVM	miR-625	Up	CMS1 and CMS3	[70]
		miR-625	Down	CMS2 and CMS4	
		miR-592	Up	CMS2	
		miR-592	Down	CMS1	
		miR-31	Up	CMS1	
	Gradient Boosting, Logistic Regression, RF	miR-1246	-	Tumor	[71]
		miR-149-3p			
		miR-4488			
		miR-607-5p			
		miR-6777-5p			
	LDA	miR-16-2-3p	Up		[72]
		miR-375-3p	Up		
		miR-378a-3p	Up		
		miR-7-5p	Up		
Hepatocellular	ANN, RF, GBC, LR	miR-221	Down	Tumor	[73]
		miR-423	Down		
		miR-26a	Down		
		miR-223	Up		
		miR-30c	Up		
		miR-365a	Up		
	RF, DT and SVM	miR-10b-5p	Up		[74]
		miR-10b-3p	Up		
		miR-224-5p	Up		
		miR-183-5p	Up		
		miR-182-5p	Up		

#### 3.4.1 Breast cancer

Identifying a minimal effective set of molecular biomarkers for cancer classification remains a major challenge in biomedical research [75]. Traditional differential expression analysis frequently generates extensive lists of dysregulated miRNAs, many of which are not clinically relevant. Sherafatian et al. introduced a novel approach using ML, specifically tree-based algorithms, to develop a small, interpretable miRNA signature for breast cancer diagnosis and molecular subtyping [66]. Among the tested models, RF demonstrated high accuracy by constructing and averaging numerous decision trees, effectively ranking key miRNAs such as hsa-miR-139, hsa-miR-183, and hsa-miR-21. Although highly accurate, the RF model is challenging to interpret because it does not clearly indicate how individual miRNAs contribute to a final decision [76]. This limits its direct applicability to clinical practice. In contrast, CART (Rpart) relies on straightforward decision rules, such as "if the expression of miR-139 is low and miR-183 is high, the sample is classified as tumor; otherwise, it is normal [77]." Using this simple logic, the model achieved comparable classification performance with just two miRNAs (hsa-miR-139 and hsa-miR-183) to distinguish tumors from normal tissue and another pair (hsa-miR-4728 and hsa-miR-190b) to classify the three major breast cancer subtypes (Luminal, HER2+, and Basal). This simplicity makes CART particularly valuable for clinical applications, as it enables physicians to implement model outcomes as straightforward diagnostic rules.

According to another investigation, three commonly used ML models—SVM, k-Nearest Neighbors (kNN), and Decision Tree (DT) —were evaluated for classifying breast cancer samples using miRNA-seq datasets [61]. SVM consistently achieved the highest accuracy due to its ability to handle high-dimensional data and define optimal separating hyperplanes, making it particularly effective for distinguishing between tumor and normal tissues. kNN also demonstrated strong performance, particularly when paired with appropriate feature selection, as it effectively captured local similarity patterns across samples. In contrast, DT showed significantly lower accuracy compared to SVM and kNN, primarily due to its tendency to overfit and its limited ability to generalize across large-scale omics data. Notably, the comparative results demonstrated that both SVM and kNN were more reliable across repeated experiments, whereas DT was the least stable. These findings indicate that SVM and kNN are more effective for miRNA-based cancer classification, while DT, despite its interpretability, may lack the predictive power needed for complex genomic datasets.

When working with imbalanced miRNA datasets, a common challenge in biomedical research where healthy controls are often limited, selecting an appropriate ML model is crucial for reliable classification. In the case of breast cancer classification, the dataset was highly skewed, with over 1100 tumor samples compared to just 104 healthy controls, making it difficult to balance sensitivity and specificity [67]. SVM model achieved nearly perfect sensitivity, but this came at the cost of specificity, as the model frequently misclassified healthy samples as cancerous due to its bias toward the majority class. In contrast, RF consistently produced high accuracy, sensitivity, and specificity in this imbalanced context. Its ensemble structure, which combines multiple decision trees trained on random subsets of both samples and features, allowed it to handle high-dimensional noise, reduce overfitting, and capture discriminative signals even from a small number of features. Furthermore, the integration of feature selection methods, such as Information Gain and Chi-Squared, enabled RF to focus on a small set of highly informative miRNAs, thereby enhancing both robustness and interpretability.

In clinical research, small datasets are a common challenge due to limited patient availability and resource constraints, making it crucial to draw conclusions from restricted sample sizes. Therefore, assessing which ML models perform optimally under such conditions is of critical significance. A recent study analyzed the circulating miRNA profiles of 66 breast cancer patients across four molecular subtypes and 16 healthy controls using a linear SVM model with 10-fold cross-validation [68]. Remarkably, the model achieved 100% accuracy for luminal A, luminal B, and triple-negative breast cancer, as well as 95% accuracy for the HER2-positive subtype, based on a small set of miRNAs. These outstanding results can be attributed to the statistical properties of the dataset: although small in sample size, each patient was characterized by the expression of 84 miRNAs, creating a high-dimensional feature space that is well-suited to SVM. By maximizing the margin between classes, linear SVM reduces model complexity and minimizes the risk of overfitting [78]. This is particularly helpful when the number of features greatly exceeds the number of samples. Furthermore, the distinct and well-separated expression signatures of the different subtypes enhanced linear separability in the feature space, allowing the algorithm to effectively discriminate between them despite heterogeneity. These findings highlight the potential of linear SVM as a powerful tool for small, high-dimensional biomedical datasets, although external validation on larger and more diverse populations is still necessary.

#### 3.4.2 Kidney cancer

Kidney cancer presents a high mortality risk, and early diagnosis remains challenging [79]. For many years, surgical intervention has been the primary treatment for kidney cancer patients. However, the prognosis is often poor, with a median survival of less than 12 months. In addition to traditional clinical methods, the investigation of various biomarkers enables researchers to gain a deeper understanding of the etiology of kidney cancer. The development of novel methods and approaches for kidney cancer detection, staging, and subtyping is crucial. MiRNAs have emerged as significant biomarkers for classifying different types and subtypes of kidney cancer [46,80].

A self-organizing deep auto-encoder deep learning model is particularly effective in classifying kidney subtypes. The model achieved remarkably high accuracy, 92% for mRNA panels and 95% for miRNA panels, along with robust AUC values across various subtypes of kidney cancer [81]. This strong performance can be attributed to the inherent ability of deep learning models to capture complex, non-linear relationships in high-dimensional transcriptomic data. The auto-encoder architecture further enabled automatic feature representation and dimensionality reduction, addressing the high-dimensionality and low-sample-size problem that commonly limits genomic studies. By integrating these properties, the deep learning framework preserves biologically meaningful patterns while filtering noise. This enhances the discriminative power of selected mRNAs and miRNAs. These advantages explain why the deep learning approach is superior to simpler algorithms and demonstrate its potential for advancing molecular subtyping in kidney cancer.

Recent investigations have shown that the application of a Long Short-Term Memory (LSTM) deep learning network is particularly effective at classifying kidney cancer [69]. The strength of LSTM lies in its ability to model complex, non-linear relationships and capture dependencies within high-dimensional miRNA expression data, which is a challenge for traditional classifiers [82]. By processing miRNA profiles in a sequential and context-aware manner, the LSTM network was able to identify subtle molecular signatures that differentiate closely related kidney cancer subtypes. This capacity explains why the model maintained strong predictive performance even when the feature space was reduced to a compact set of 35 discriminative miRNAs, demonstrating that LSTM is not only effective in handling large-scale genomic data but also resilient in lower-dimensional scenarios. However, despite the promising results of deep learning for cancer molecular subtyping, certain limitations remain. LSTM models generally require large and balanced datasets to prevent overfitting [83]. While data augmentation was used to address imbalance, synthetic approaches may not fully capture the biological diversity present in the data. Furthermore, the computational complexity and limited interpretability of deep neural networks present challenges for clinical translation, as physicians may require transparent explanations for subtype predictions.

Advanced approaches that integrate deep learning with the interpretability of fuzzy logic have recently emerged for kidney cancer classification based on miRNA profiles [46]. The proposed self-organizing deep neuro-fuzzy system exemplifies this trend by combining the transparency of fuzzy rule-based reasoning with the capacity of deep architectures to model complex nonlinear interactions. Unlike conventional neuro-fuzzy models, such as ANFIS, where the number of fuzzy rules increases exponentially with the number of features, this novel design employs a modified fuzzification and rule layer that reduces the growth to a linear scale, addressing the curse of dimensionality in high-throughput genomic data [84]. Furthermore, by incorporating a self-organizing deep autoencoder within the rule layer, the system generates higher-order rules from simple ones, enhancing its ability to capture intricate dependencies across miRNAs. A key strength of this architecture is its robustness to class imbalance and small sample sizes, enabling reliable classification without the need for artificial data augmentation. At the same time, its fuzzy logic foundation preserves interpretability, allowing the derivation of clinically meaningful rules. Taken together, this hybrid framework provides a powerful and transparent tool for accurate kidney cancer subtyping in the context of miRNA-driven diagnostics.

#### 3.4.3 Colorectal cancer

Colorectal cancer remains one of the leading causes of cancer-related morbidity and mortality worldwide, highlighting the urgent need for reliable, non-invasive diagnostic tools that enable early detection [85]. By combining fecal miRNA signatures with ML algorithms, colorectal cancer can be accurately distinguished from controls. Instead of most previous investigations that relied on blood or tumor-derived material, the authors focused on fecal samples as the primary source of miRNAs, offering a practical and patient-friendly strategy for early cancer screening [71]. Through a comprehensive ML pipeline, they reduced thousands of candidates derived from small RNA sequencing to a robust five-miRNA signature that achieved exceptionally high diagnostic performance (AUC up to 0.96) in independent validation cohorts. The performance of the LDA-based model in recent evaluations confirms the potential of fecal-derived miRNA signatures for the classification of colorectal cancer [72]. Linear Discriminant Analysis (LDA) is a well-established supervised learning technique designed to identify a linear combination of features that maximizes the differentiation between two or more predefined groups [86]. By contrast to complex "black-box" algorithms, LDA focuses on maximizing the ratio of between-class variance to within-class variance, effectively projecting the data into a space where class distinctions are most evident. This framework was applied to fecal-derived miRNA profiles, enabling the extraction of a small set of highly discriminative features. By refining the expression data through feature engineering and integrating four upregulated miRNAs into the model, LDA constructed a composite score that robustly differentiated colorectal cancer patients from healthy controls. The results were striking, with the model achieving near-perfect diagnostic performance (AUC >0.98 in the training cohort and >0.94 in the validation cohort), outperforming conventional serum biomarkers. These findings demonstrate not only the biological informativeness of miRNA signatures but also the power of LDA to transform them into clinically meaningful classifiers, capable of clearly distinguishing between diseased and normal states with high sensitivity and specificity.

The study by Guinney et al. highlights the application of a RF classifier to categorize Colorectal cancer samples into consensus molecular subtypes (CMSs), using miRNA expression profiles as a key input feature. It is a classification system for colorectal cancer based on gene expression patterns [87]. The CMSs divide colorectal cancer into four subtypes, each with different biological and clinical characteristics: CMS1, which has a high mutation rate, a strong immune response, and a good prognosis; CMS2, which has a typical epithelial appearance, with activated WNT and MYC signaling pathways; CMS3, which has a metabolic dysregulation, with altered glucose and lipid metabolism; and CMS4, which has a mesenchymal phenotype, with high levels of stromal invasion and angiogenesis. The RF and SVM classifiers were used to distinguish CMS-associated types based on miRNA levels [70]. The models were trained and tested on various colorectal cancer miRNA-seq datasets obtained from TCGA. The results showed that the RF model has more accuracy compared with the SVM. Besides, the most relevant and significant miRNAs that correlate with each CMSs subtype are summarized in Table 3.

#### 3.4.4 Hepatocellular carcinoma

MiRNA expression profiles from bodily fluids, combined with ML algorithms, differentiate hepatocellular carcinoma (HCC) from liver metastasis. A key challenge in liver cancer diagnosis is the overlap in clinical and imaging features between HCC and liver metastasis [88]. By combining miRNA biomarkers and advanced ML techniques, early detection can be enabled, leading to timely treatment. Various ML models for analyzing miRNA data and assessing their effectiveness in distinguishing HCC from liver metastasis have been evaluated. RF emerged as the top-performing model in evaluating ML algorithms for distinguishing HCC from liver metastasis [73]. Compared to Artificial Neural Networks (ANN), Gradient Boosting Classifier (GBC), and Logistic Regression (LR), RF demonstrated superior accuracy and reliability. RF achieved 91% accuracy and an AUC exceeding 0.95, enabling precise differentiation between the two classes. In contrast, ANN and Logistic Regression showed significantly lower accuracy, particularly in cases with limited or imbalanced data. RF's ability to handle complex datasets ensured consistent and reliable performance. The study highlights the benefits of optimizing ML models by incorporating sample quality parameters, such as RNA concentration and other relevant metrics. This will significantly improve predictive accuracy, where alternative diagnostic methods are essential. ML models, especially RF, show significant potential not only for distinguishing HCC from liver metastasis but also as effective, non-invasive tools for early detection and treatment of liver cancers in clinical practice. A study on the differentiation of HCC from the control group using miRNA profiles with various ML models identified RF as the best-performing model [74]. RF's superior performance stems from its ability to classify data and identify significant features from thousands of miRNAs. By leveraging complex features, RF achieved the highest AUC value (98.2%), effectively distinguishing between cancerous and normal samples. Additionally, RF's capacity to handle imbalanced and nonlinear data contributed to its outperformance compared to other models, such as SVM and DT.

## 4 Conclusion

Detecting cancer at an early stage remains a significant challenge, driving extensive research in this field. Biomarkers, particularly those associated with cancer, have shown promise in early detection. To reduce drug side effects and improve patient outcomes, researchers are focused on developing methods for accurate cancer diagnosis and classification of cancer types and subtypes. Much of this work relies on cancer-specific RNA expression data, such as miRNA-seq. MiRNAs are recognized as potential biomarkers for various cancers, but further research is needed to understand how miRNA profiles can enhance clinical classification. Currently, there is no consensus on the optimal number or combination of miRNAs to serve as reliable biomarkers. Additional studies are required to explore the diversity and complexity of miRNA expression across different cancers and to establish standardized, robust methods for miRNA detection and analysis. ML algorithms, particularly in real-time systems, have the potential to significantly improve cancer diagnosis and treatment outcomes by enabling rapid assessment and decision support using miRNA-seq datasets. Various ML models have been developed to distinguish between cancer types and tumor subtypes, with researchers also creating multi-cancer classification models with high accuracy. Furthermore, integrating miRNA data with other molecular data, such as genomic, transcriptomic, proteomic, and epigenetic data, could provide a more comprehensive understanding of the molecular mechanisms and pathways involved in cancer development and progression. Future studies must address several limitations, including dataset imbalance, batch effects, and reproducibility, all of which require careful consideration. Dataset imbalance can lead to biased model predictions, batch effects can cause unwanted variations in results, and ensuring reproducibility is essential for validating findings and enabling their reliable application across different datasets and research contexts.

## Data sharing statement

Hereby we state that data sharing is not applicable in our submission.

## Funding

This research received no specific grant from any funding agency in the public, commercial, or not-for-profit sectors.

## CRediT authorship contribution statement

**Zeinab Tariri:** Conceptualization, Methodology, Writing – original draft. **Mehdi Goodarzi:** Conceptualization, Data curation, Writing – original draft. **Atieh Nouralishahi:** Conceptualization, Validation, Writing – original draft. **Malihe Sagheb Ray Shirazi:** Conceptualization, Investigation, Writing – original draft. **Meysam Mohammadikhah:** Conceptualization, Writing – review & editing. **Azita Sadeghzade:** Conceptualization, Writing – review & editing. **Hossein Gandomkar:** Conceptualization, Supervision, Writing – review & editing. **Ehsan Maghrebi-Ghojogh:** Conceptualization, Supervision, Writing – review & editing.

## Declaration of competing interest

There is no conflict of interests.

## Data Availability

No data was used for the research described in the article.
